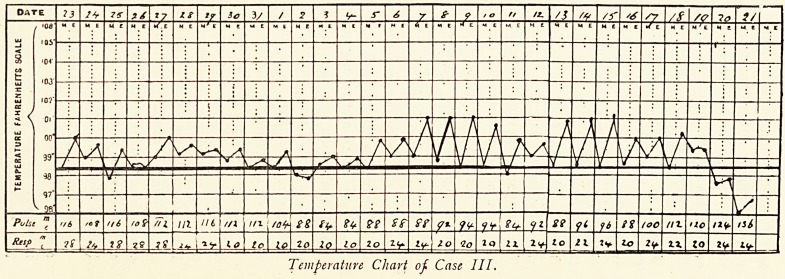# Aleucocythæmic Leukæmia

**Published:** 1913-03

**Authors:** Rupert Waterhouse

**Affiliations:** Physician to the Royal Mineral Water Hospital; Pathologist and Assistant Physician to the Royal United Hospital, Bath.


### ALEUCOCYTHÆMIC LEUKÆMIA.

BY

RUPERT WATERHOUSE, M.D. Lond., M.R.C.P.

*Physician to the Royal Mineral Water Hospital;*
*Pathologist and Assistant Physician to the Royal United Hospital, Bath.*

---

THE name leukæmia, and even more so that of leucocythæmia, implies that the very essence of the disease consists in an increase in the number of white cells in the blood. When this increase affects the granular cells, and their precursors, myelocytes, appear in large numbers in the circulation, the condition is known as myelæmia, myelocythæmia, or myeloid, myelogenous, or spleno-medullary leukæmia. When, on the other hand, the increase affects the non-granular cells, the names lymphæmia, lymphocythæmia, or lymphatic leukæmia are applied.

For some time it has been known that in leukæmia, under certain circumstances, the number of leucocytes in the blood may fall to or even below normal. This is seen especially as the result of treatment by X-rays, tuberculin, arsenic, etc., and as the effect of intercurrent diseases, such as influenza, typhoid fever, and septic complications. Cabot[1], indeed, records a case of lymphatic leukæmia in which the number of cells dropped as the result of suppuration occurring in the cervical glands from 40,000 per cubic millimetre to a tenth of this number, and finally, on the day of death, to the extraordinarily small number of 471 per c.mm.

Speaking generally, it may be said that in lymphatic leukæmia the more chronic the disorder the greater the number of leucocytes in the blood. In chronic cases these cells usually number from one to two hundred thousand per cubic millimetre. It is difficult to name an average for the acute cases since, as Muir[2] points out, many so-called acute cases are in reality merely the acute terminations of chronic ones. There is no doubt, however, that in many of the acute cases the number is quite small. Thus Gulland and Goodall[3] say: "We have several times made the diagnosis with counts as low as 10,000, and once with a count of 6,800, and have seen these cases die with counts of 17,500, 19,000, 20,000, and 25,000."

In the series of cases in children brought before the Royal Society of Medicine in 1908 by Forbes and Langmead[4], the counts were in some instances even lower than this. These writers came to the conclusion that "in the more acute cases (of lymphocythæmia), when the lymphocyte count is highest there is a greater numerical increase both relatively and absolutely of the large lymphocytes than of the small, and at this time death may occur. In the more prolonged cases, as this period is passed there is a marked decline in the total number of lymphocytes, affecting the small lymphocytes relatively less than the large, and the fall may be so considerable as to result in a leucopenia immediately preceding death." That this does not always hold good in adults is shown by one of the cases about to be described, in which a previously low count was followed by a flooding of the blood with lymphocytes just before death, and many similar cases have been recorded which formerly, before the distinction between the histological appearances in leukæmia and in lymphadenoma had been made, led to the supposition that the latter disease might become converted into the former.

One interesting point about these cases with low counts lies in the difficulty of diagnosis. As a rule, this difficulty is largely discounted by the presence of the great relative increase of the lymphocytes, which usually number over 90 per cent. As will be seen, however (Case 3), their percentage may at times be considerably less than this. Nevertheless, on the whole, the combination of the clinical symptoms and the blood findings presents a picture which is very characteristic.

**Case 1.** [1]—A mentally-deficient boy, aged 20, the subject of ichthyosis since birth, in September, 1906, complained of pains in his limbs, which continued, on and off to the time of his death, six months later. In December he had a severe attack of epistaxis. In January, 1907, the glands in the neck enlarged. In February he had another attack of epistaxis, and on March 16th was admitted to the Royal United Hospital under the care of Dr. Bannatyne. He was profoundly anæmic. The glands of the neck, axillae and groins were enlarged, as was also the spleen. Both knee-joints contained fluid. He gradually became weaker, and died somewhat suddenly after a brief period of restlessness and dyspnœa a fortnight after admission.

BLOOD EXAMINATIONS.

| 1907. | Erythro-cytes. | Erythro-blasts. | Leuco-cytes. | Polymorpho-nuclears. | Lympho-cytes. | Myelo-cytes. |
|---|---|---|---|---|---|---|
| Mar. 17. | 1,152,000 | — | 6,200 | — | — | — |
| Mar. 18. | 1,184,000 | 40 | 6,000 | 720 | 5,240 | 40 |
| Mar. 26. | 1,107,000 | 12 | 7,000 | 606 | 6,356 | 12 |

*Temperature Chart of Case I.*

[1] This case is fully reported in the *Lancet*, 1908, vol. ii. p. 869, and will be only briefly summarised here.

**Case 2.**—Male, aged one year and eight months. Breast fed, and except for a tendency to bronchitis, healthy up to the age of six months, when it was said to be " rickety." Mother has three other children, all healthy, and has had no miscarriages. Child was always pale. Glands in neck said to be enlarged since eight months old. On October 23rd, 1909, had an attack of epistaxis, and a week later was admitted to the Royal United Hospital under Dr. Bannatyne. Child was profoundly anæmic, but bright and intelligent for its age. Enlarged glands the size of marbles—visible, firm, discrete—could be felt under the angle of the jaw on the right side, and chains of pea-like glands behind either sternomastoid in the axillae and in the groins. The liver was enlarged to the umbilicus, and the spleen could be felt one inch below the ribs. There was a bruise on the left thigh the size of a shilling, and the child had been noticed to bruise very readily. No retinal hemorrhages. Death occurred on November 5th, preceded by restlessness and dyspnœa.

BLOOD EXAMINATIONS.

| 1909. | Erythro-cytes. | Erythro-blasts. | Hæmo-globin. | Colour Index. | Leuco-cytes. | Poly-morpho-nuclears. | Lympho-cytes. |
|---|---|---|---|---|---|---|---|
| Oct. 30. | 1,333,000 | — | — | — | 9,800 | — | — |
| Oct. 31. | 1,642,000 | 0 | 25 % | .83 | 10,600 | 800 | 9,800 |
| Nov. 1. | 1,400,000 | — | 21 % | .75 | 12,800 | — | — |
| Nov. 3. | 960,000 | — | 18 % | .94 | 8,600 | — | — |

*Temperature Chart of Case II.*

**Case 3.**—A platelayer, aged 26, was perfectly well until July, 1910, when he began to suffer from symptoms of anæmia, and the glands of his neck swelled. On August 22nd he was admitted to the Royal United Hospital under the care of Dr. Wilson-Smith. He was profoundly anæmic. The axillary glands were the size of peas, those in the groins rather larger, whilst the cervical glands were distinctly enlarged, firm and discrete, the largest the size of a bantam's egg. There were petechiæ on the posterior wall of the pharynx, and especially on the posterior pillars of the fauces, whilst the tonsils, swollen and unhealthy-looking, presented a curious blotchy, plum-coloured appearance. The breath was very fœtid. There were a few petechiæ in the skin of the lower part of the abdomen, and numerous retinal hemorrhages in the neighbourhood of and radiating from both optic discs. There were hæmic murmurs ovei the heart, and the spleen could just be felt below the costal margin. Urine acid 1,015, contained neither albumin nor sugar, but deposited large quantities of uric acid crystals. *August 24th.*—Vomited twice. *August 25th.*—Salol gr. x. t.d. prescribed. *August 27th.*—Daily insufflation of sulphur to the back of the throat begun. *August 30th.*—Lymphatic glands less than half their size on admission. Salol changed to naphthalene tetrachloride, gr. viii. every four hours. *September 6th.*—Striking improvement during the last week in colour and general appearance ; glands much smaller ; throat practically normal ; fœtor of breath gone ; spleen has not been palpable since day of admission. *September 8th.*—Had some diarrhœa last night, so insufflation of sulphur discontinued. Spleen distinctly felt to-day. *September 16th.*—Both spleen and glands have gradually increased in size during the last week. *September 17th.*—Severe attack of epistaxis. *September 20th.*—Spleen smaller, but cervical glands much enlarged. More epistaxis ; very severe ; vomiting semi-digested blood ; melæna. Nose plugged. *September 21st.*—Died.

BLOOD EXAMINATIONS.

| 1910. | Erythro-cytes. | Erythro-blasts. | Hæmo-globin. | Co-lour Index. | Leuco-cytes. | Poly-morpho-nuclears. | Lympho-cytes. | Myelo-cytes. |
|---|---|---|---|---|---|---|---|---|
| Aug. 22. | 1,573,000 | 300 | — | — | 11,300 | 1,300 | 10,000 | — |
| Aug. 25. | 939,000 | 400 | 16 % | .86 | 4,100 | 1,940 | 2,370 | — |
| Aug. 28. | 1,027,000 | 120 | 18 % | .87 | 3,000 | 890 | 2,130 | — |
| Aug. 31. | 1,354,000 | 150 | 26 % | .96 | 3,650 | 1,850 | 1,800 | — |
| Sept. 4. | 2,876,000 | 300 | 45 % | .78 | 6,740 | 2,960 | 3,420 | 360 |
| Sept. 11. | 2,950,000 | — | 38 % | .64 | — | — | — | — |
| Sept. 19. | 1,180,000 | — | 26 % | 1.1 | 83,000 | — | — | — |
| Sept. 20. | — | None. | — | — | 108,000 | 3,600 | 104,400 | None. |

The symptoms common to these three cases may be summarised as follows :—

(1) *Profound anæmia.*

(2) *Hemorrhages,* especially epistaxis.

(3) *Irregular pyrexia.*

(4) *Enlarged glands,* the cervical being specially affected.

(5) *Slight enlargement of the spleen.*

The blood in all cases was pale and watery but clotted rapidly. The red-blood cells numbered about a million and a half, the hæmoglobin being diminished but little more in proportion, so that the colour index was only just below normal. The size of the red-blood cells in any one film sometimes varied a good deal ; sometimes, as for instance during the period of improvement in Case 3, but little. Polychromatophilia was common, basophil granulations absent, poikilocytosis never extreme, blood platelets very scanty. Both megaloblasts and normoblasts were present in Cases 1

*Temperature Chart of Case III.*

and 3, but the commonest nucleated red cell, judged by its size and the staining intensity of its nucleus, was something between the two. Some of these were seen undergoing division, and some extruding their nucleus.

As regards the leucocytes, it will be observed that the count varies between 3,000 and 11,300, except that in Case 3, just before death the blood became crowded with lymphocytes. Basophils (with one or two doubtful exceptions) and eosinophils were absent in all films examined. The polymorphonuclear cells were diminished in number in every instance, and generally very markedly. The nuclei of these generally showed but two or three lobes, seldom four or five. The lymphocytes were generally about normal in number, but were sometimes greatly increased. There was always a relative increase. No attempt has been made to differentiate between the small and the large lymphocytes for the reason that every gradation was met with between typical small and typical large cells, whilst those most frequently encountered were about midway between the two, namely about the size of a polymorphonuclear cell. In all cases these cells were abnormally fragile and readily burst, so that it was much more difficult than usual to obtain satisfactory preparations. Their protoplasm sometimes contained a few azurophil granules. *Post-mortem* examinations in all these cases disclosed lesions typical of lymphatic leukæmia.

*The bone marrow* of the shafts of the tibiæ was deep red and gelatinous in Cases 2 and 3, and in Case 1 pale pink, with patches of white brain-like deposit and a few small hemorrhages. Films showed lymphocytes and erythrocytes in about equal numbers in all cases. The only other cells seen were a few eosinophil cells (not more than one to a thousand lymphocytes), and in Case 2 a few very large non-granular cells like giant lymphocytes.

*The lymphatic glands.*—The axillary, the inguinal, and especially the cervical glands, were enlarged in all cases. The mesenteric glands were enlarged in Cases 2 and 3, not in Case 1. The mediastinal and portal glands were enlarged in Case 1, but are not mentioned in the others. Microscopically all distinction between cortex and medulla was lost, the glands being crowded with lymphocytes.

*The thymus* was not persistent in Case 1, is not mentioned in Case 3, and in the child (Case 2) was pale, not enlarged, microscopically showed large numbers of Hassall's corpuscles, and appeared normal.

*The lungs.*—Scattered sub-pleural petechiæ were present in Cases 2 and 3, and in Case 1 the lower lobes were red, congested and gelatinous.

*The heart* exhibited marked fatty degeneration (thrush's breast heart) in the case of the child. In Case 3 it was of a pale chocolate colour, and showed sub-pericardial petechiæ. None of the cases showed lymphocytic infiltrations microscopically.

*The liver* weighed 66 ounces in Case 1, 80 ounces in Case 3, and 16 ounces in the child. Every case showed large collections of lymphocytes, resembling those seen in the blood, in the portal spaces. Case 1 gave a fairly well-marked Prussian blue reaction.

*The spleen* weighed 14 ounces in Case 1, 21 ounces in Case 3, and 1¾ ounces in the child. There were small hemorrhages on the surface, and hemorrhages of older date in the substance in Case 3. Microscopically the pulp was packed with lymphocytes. The Malpighian bodies were distinct in Case 2, not in the others.

*The kidneys* were very pale in all instances, but in Case 3 blotchy from subcapsular hemorrhages. Microscopical changes were very pronounced in all three, consisting of collections of lymphocytes in masses under the capsule and in the cortex, and passing down in the form of strands between the tubules in the medulla. In Case 1 the right kidney was absent, the left weighed 11 ounces. In Case 3 they weighed 15 ounces and 13 ounces respectively.

*The adrenals.*—In Case 1 the right suprarenal showed a few white patches in the medulla. The medulla of the left was completely transformed into white leukæmic material. The condition of the adrenals in the other cases is not mentioned.

*The intestines* showed enlargement of the solitary follicles, and lymphocytic infiltrations amongst the crypts, and in Case 3 small hemorrhages.

*The thyroid and pancreas* were normal, naked eye and microscopically.

*The synovial membrane* of the knee-joints in Case 1 was soft, thickened and injected, and the synovia was increased in amount. Microscopically, beneath the endothelium was a sharply-defined continuous layer of lymphocytic deposit, rather more than 50 $\mu$ in depth. In the deeper layers of the membrane were scattered patches of lymphocytes around the blood vessels.

### REFERENCES.

[1] *A Guide to the Clinical Examination of the Blood,* 2nd Ed., 1897.

[2] *A System of Medicine,* Allbutt and Rolleston, Vol. v., 1909.

[3] *The Blood,* Gulland and Goodall, 1912, p. 181.

[4] *Proceedings of the Royal Society of Medicine,* Vol. i., Pt. i., Clinical Section, p. 162.

## Figures and Tables

**Figure f1:**
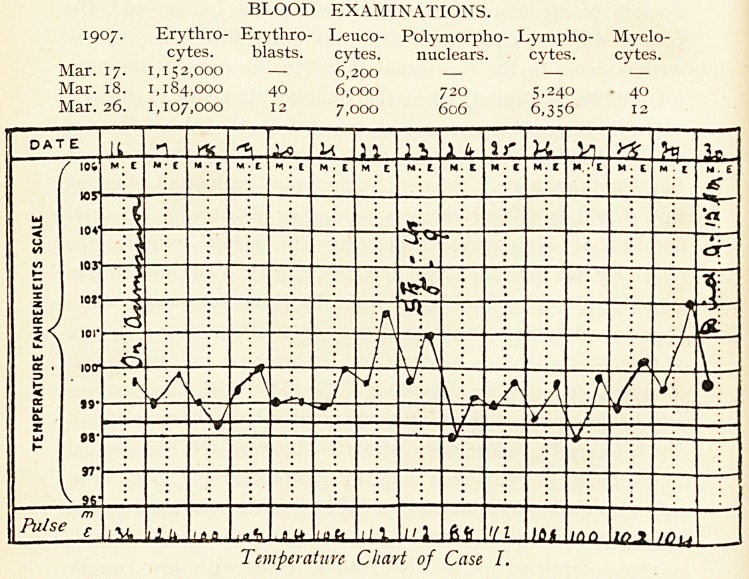


**Figure f2:**
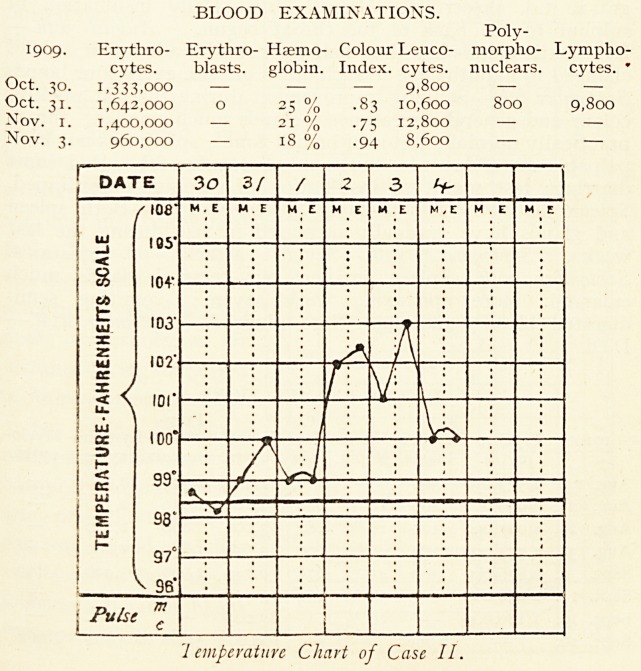


**Figure f3:**